# Biocomposites of Bio-Polyethylene Reinforced with a Hydrothermal-Alkaline Sugarcane Bagasse Pulp and Coupled with a Bio-Based Compatibilizer

**DOI:** 10.3390/molecules25092158

**Published:** 2020-05-05

**Authors:** Nanci Vanesa Ehman, Diana Ita-Nagy, Fernando Esteban Felissia, María Evangelina Vallejos, Isabel Quispe, María Cristina Area, Gary Chinga-Carrasco

**Affiliations:** 1 IMAM, UNaM, CONICET, FCEQYN, Programa de Celulosa y Papel (PROCYP), Misiones, Félix de Azara 1552, Posadas, Argentina; ffelissia@gmail.com (F.E.F.); mariaxvallejos@gmail.com (M.E.V.); cristinaarea@gmail.com (M.C.A.); 2 Peruvian LCA and Industrial Ecology Network (PELCAN), Department of Engineering, Pontificia Universidad Católica del Perú (PUCP), 1801 Avenida Universitaria, San Miguel, Lima 15088, Peru; dita@pucp.edu.pe (D.I.-N.); iquispe@pucp.edu.pe (I.Q.); 3 RISE PFI, NO-7491 Trondheim, Norway

**Keywords:** bio-based filament, 3D printing, sugarcane bagasse pulp

## Abstract

Bio-polyethylene (BioPE, derived from sugarcane), sugarcane bagasse pulp, and two compatibilizers (fossil and bio-based), were used to manufacture biocomposite filaments for 3D printing. Biocomposite filaments were manufactured and characterized in detail, including measurement of water absorption, mechanical properties, thermal stability and decomposition temperature (thermo-gravimetric analysis (TGA)). Differential scanning calorimetry (DSC) was performed to measure the glass transition temperature (Tg). Scanning electron microscopy (SEM) was applied to assess the fracture area of the filaments after mechanical testing. Increases of up to 10% in water absorption were measured for the samples with 40 wt% fibers and the fossil compatibilizer. The mechanical properties were improved by increasing the fraction of bagasse fibers from 0% to 20% and 40%. The suitability of the biocomposite filaments was tested for 3D printing, and some shapes were printed as demonstrators. Importantly, in a cradle-to-gate life cycle analysis of the biocomposites, we demonstrated that replacing fossil compatibilizer with a bio-based compatibilizer contributes to a reduction in CO_2_-eq emissions, and an increase in CO_2_ capture, achieving a CO_2_-eq storage of 2.12 kg CO_2_ eq/kg for the biocomposite containing 40% bagasse fibers and 6% bio-based compatibilizer.

## 1. Introduction

Three-dimensional (3D) printing allows the manufacturing of custom pieces that usually demand higher costs and production time when manufactured by conventional methods. In addition, it offers unparalleled flexibility in achieving controlled composition, geometric shape, functions, and complexity [1]. Numerous studies about ink formulations for 3D printing have shown that bioplastics are promising materials for 3D printing applications [2,3,4,5,6].

Bioplastics are obtained from first-generation (1G) resources (starches or sugars, such as corn, sugarcane, wheat, and soy) or second-generation (2G) resources (cellulose from crops or industrial processes). One of the bioplastics that are expected to grow greatly in the next years is bio-polyethylene (BioPE) 1G for flexible and rigid packaging applications [7].

The main advantage of BioPE with respect to traditional polyethylene (PE) (fossil-based) is the utilization of renewable raw materials to reduce greenhouse gas emissions [8]. However, BioPE has relatively low mechanical properties (modulus and strength) compared to other commodity materials. Hence, the inclusion of dispersed phases (e.g., cellulosic fibers) with high stiffness and strength is commonly applied to enhance its mechanical properties [9]. Studies evaluating the effect of natural fibers include raw materials such as wood sawdust, bleached and unbleached pine pulps [10], eucalyptus pulp [11], and nonwoods such as bamboo fibers [12], flax [13] and kenaf [14]. The previous studies showed that the addition of fibers or pulps increased the tensile strength and Young’s modulus and caused some variations in thermal properties.

**Figure 1:**
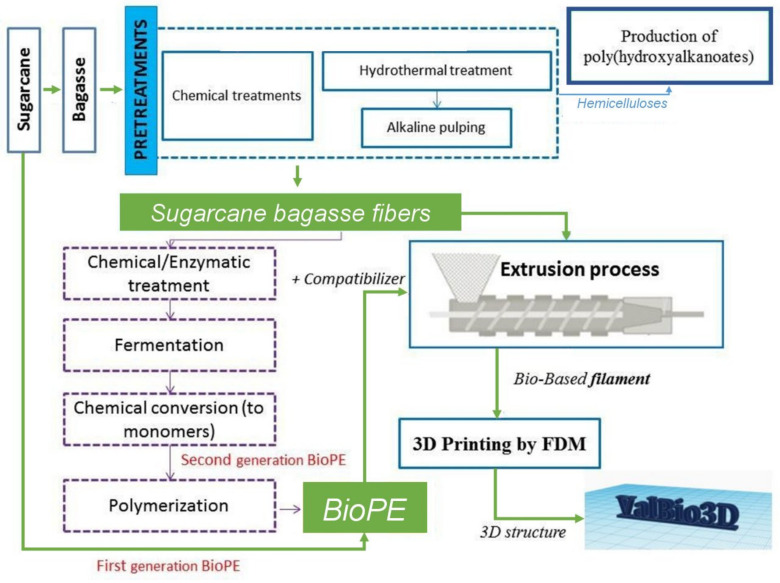
Scheme for the integral use of sugarcane, including the production of Bio-polyethylene (BioPE) and bagasse fibers.

Sugarcane bagasse is a lignocellulosic agro-industrial waste generated by the sugar and alcohol industries and is usually burned in the sugar mill. The valorization of sugarcane bagasse implies its integral use in a lignocellulosic biorefinery scheme [15]. Figure 1 shows different routes to obtain products with an integrated approach concept. Hydrothermal treatment allowed the extraction of the hemicelluloses fraction to produce polyhydroxialkanoates (PHAs) [16], which are a group of bioplastics. The solid fraction after treatments contains sugarcane bagasse fibers. Fibers can take two alternative routes: they can be subjected to an enzymatic treatment followed by fermentation, chemical conversion, and finally, polymerization to obtain second generation BioPE [17], or they can be used as reinforcement for biocomposites and 3D printing [18].

A good interfacial bonding is required to achieve optimum reinforcement. Hence, the bonding between fibers and matrix plays a vital role in determining the mechanical properties of the biocomposites. For plant-based fiber composites, there is limited interaction between the hydrophilic fiber and the usually hydrophobic matrices, limiting their mechanical performance [19]. This effect could be reduced by introducing a compatibilizer. The most common compatibilizer used for the combination of BioPE with fibers is the maleic anhydride (MA). MA is grafted on polyethylene (PE) to form maleate polyethylene (MAPE), which is commercialized as a compatibilizer. MAPE covalently couples PE and fibers [20]. Studies that included 5 wt% of MAPE to coupling PE with bamboo fibers showed increases in flexural and tensile strength [21], respect to the composite without compatibilizer. Yuan et al. [22] used only 3 wt% of MAPE as compatibilizer between PE and fibers of maple wood and found that the use of the coupling agent increased the tensile strength. Tarrés et al. [23] found an optimal load of 6 wt% of MAPE to maximize the tensile properties of BioPE reinforced with thermo-mechanical pulp (TMP) fibers. Additionally, Mendez et al. [24] used the same load of MA-grafted polypropylene to evaluate the mechanical properties of polypropylene reinforced with groundwood pulp from pine. However, MAPE is conventionally derived from fossil resources; therefore, it does not contribute to obtaining a 100% bio-based product.

Recent studies have evaluated filaments of BioPE and wood fibers for 3D printing applications. Filgueira et al. [18] obtained filaments of BioPE with TMP fibers modified enzymatically. This approach reduced the water absorption of the filaments and improved the 3D printability of structures. Similarly, Tarrés et al. [23] obtained 3D structures by 3D printing (Fused Deposition Modeling, FDM) of BioPE biocomposites, evaluating the impact of different TMP fiber loads on the mechanical properties.

This work aims to assess the potential advantages of biocomposites filaments containing BioPE, sugarcane bagasse pulp and a bio-based compatibilizer (Figure 1), compared to neat BioPE or in combination with a fossil-based compatibilizer. For filaments composed of BioPE, two percentages of pulp fibers and two compatibilizers (fossil and bio-based) were manufactured. Mechanical properties and water absorption of the filaments were determined. Scanning electron microscopy (SEM) images from the fracture area of the tensile tested filaments were acquired. The thermal stability and decomposition temperature of the BioPE and biocomposites were determined with thermo-gravimetric analysis (TGA) and differential scanning calorimetry (DSC) and the glass transition temperature (Tg) was measured. The capability of the filaments to be extruded and deposited on a substrate to form a 3D shape was evaluated and demonstrated. Importantly, we performed a cradle-to-gate study to assess the impacts of replacing the fossil-based compatibilizer with a bio-based alternative.

## 2. Results

The chemical composition of the raw material and pulp are shown in Table 1.

**Figure 2:**
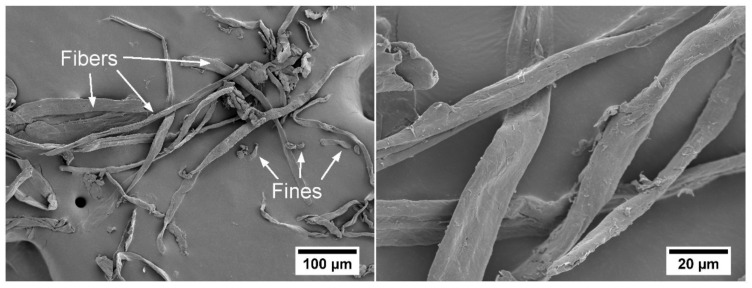
SEM images of HT/Soda fibers. Left) Fibers and fines are exemplified (magnification 200×). Right) Fibers observed at 1000× magnification.

Bagasse, remaining from BioPE production, can be used to obtain sugarcane bagasse pulp, which can be further used to reinforce BioPE and thus close the loop in a biorefinery. In this study we applied a hydrothermal treatment (HT)/Soda treatment on the raw material to allow the release of the lignocellulosic fibers (HT/Soda fibers). Examples of milled HT/Soda fibers are provided in Figure 2. The quantification of the fiber morphology with a Fiber Tester device revealed an average fiber length of 367 μm, fiber width of 24.3 μm and a fraction of fines of 50%.

Bagasse, remaining from BioPE production, can be used to obtain sugarcane bagasse pulp, which can be further used to reinforce BioPE and thus close the loop in a biorefinery. In this study we applied an HT/Soda treatment on the raw material to allow the release of the lignocellulosic fibers (HT/Soda fibers). Examples of milled HT/Soda fibers are provided in Figure 2. The quantification of the fiber morphology with a Fiber Tester device revealed an average fiber length of 367 μm, fiber width of 24.3 μm and a fraction of fines of 50%.

The glucans content increased from 40.4% oven dry (od) material to 90.8% od pulp after the HT/Soda treatments. The hemicelluloses are mainly eliminated during hydrothermal stage from 35.0% od material to 5.1% od pulp [25], whilst during alkaline treatment lignin is mainly dissolved, which is then eliminated with pulp liquor (from 20.6% to 2.1% od). HT/Soda pulp total yield and kappa number were 32.4% and 16.8%, respectively.

The HT/Soda fibers were compounded with BioPE and the corresponding compatibilizers to form filaments for 3D printing (Figure S1). Various properties of the filaments were assessed as we will explore in the next sections.

### 2.1. Water Absorption Behavior

**Figure 3:**
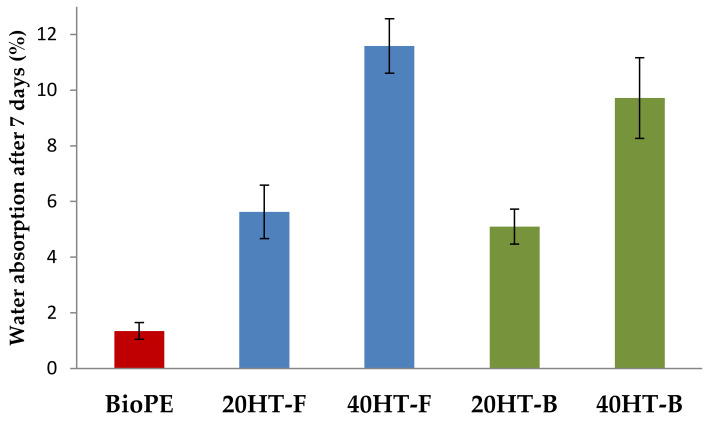
Water absorption of filaments with different percentages of fibers, using fossil- and bio-based compatibilizers.

Water absorption was determined by immersing the filaments in water for 7 days (Figure 3). The importance of this analysis is that the exposure of cellulose-reinforced biocomposites to moisture can cause the cellulosic elements to swell, a phenomenon that can lead to the weakening of the structure [26]. Several authors have studied the effect of hemicellulose content on water absorption and found that reducing the amount of hemicelluloses reduces the swelling capacity of the fibers [27,28,29,30]. The chemical composition for HT/Soda pulp used as reinforcement showed low hemicellulose content. Therefore, the increase in water absorption was expected to be relatively low.

ANOVA analysis indicated significant differences in water absorption values in respect to the fiber load (*p* < 0.05). The increase in fiber content in the filaments caused an increase in water absorption due to the hydrophilic nature of the fiber. Compared to the BioPE filament, the samples containing 20% and 40% fiber showed an increase in water absorption of approx. 6% and 10%, irrespective of the used compatibilizer. It is worth mentioning that the fibers applied in this study were not modified to increase their hydrophobicity. Thus, the increase in fiber content produces an increase in water absorption due to the hydrophilic and hygroscopic nature of the fibers used as reinforcement.

Previously, we have demonstrated that using laccase enzymes to graft gallate compounds on the surface of lignocellulosic fibers could reduce the water absorption considerably [18].

An interesting feature observed was the presence of bubbles on the surface of all the fiber-containing filaments after 24 h of immersion in water. The presence of bubbles can be due to the roughness of the filament surface that generates empty spaces containing air, which is displaced by water. The effect of the filament roughness on the water absorption was previously demonstrated by Filgueira et al. [18], who found that a high filament roughness leads to a high specific surface area that increases the contact between the filament and water.

### 2.2. Mechanical Properties

The addition of fibers as reinforcement in polymer matrices yields an increase in mechanical properties, such as strength and stiffness. The mechanical properties of the filaments are presented in Table 2.

Tensile properties provide useful information about the microstructure and the interface between the different materials [23]. The filaments with 40 wt% fibers presented the highest tensile strength, which was significantly different (*p* < 0.01) compared to the neat BioPE and the sample with 20% fiber fraction. For the two compatibilizers, increments of roughly 20% and 60% were found for fiber loads of 20 wt% and 40 wt%, respectively. Higher values of tensile strength were found by other authors using TMP fibers, reaching increments of up to 74%, adding 20% of fibers [23]. It was presumed that the quantified difference in tensile strength between our study and Tarrés et al. [19] was due to the length of the TMP and bagasse fibers. In this study the bagasse fibers were milled in order to ease the blending of the fibers and BioPE, with the equipment used in this study (Figure 2). However, Tarrés et al. [19] quantified the length of the TMP fibers in the biocomposite, and this was in the same range as the sugarcane bagasse fibers used in this study (~200–400 µm). Hence, the higher value in tensile strength obtained by Tarrés et al. [19] may be due to two factors: i) the morphology of the TMP fibers which are more fibrillated (larger fraction of split fibers) compared to the more intact chemical pulp fibers [31] and ii) a more effective compounding which probably leads to a more homogeneous fiber spatial distribution. It is expected that the more fibrillated TMP fibers have a large surface area which may facilitate the anchoring of the fibers in the BioPE matrix. Additionally, keep in mind that the mechanical testing in the present study was performed on filaments, while the quantification performed by Tarrés et al. [19] was based on injection molding samples. This is also expected to affect the mechanical performance of the biocomposite materials.

Similar to tensile strength, Young’s modulus increased when increasing fiber loadings. Fibers are located between the polymer chains of the BioPE, reducing their mobility [24], and thus increasing the stiffness. The increment in stiffness with the addition of 20 wt% and 40 wt% fibers and both compatibilizers was about 25% and 75% compared to neat BioPE, respectively. The strain at break for all the produced biocomposites decreased with respect to the BioPE filament. An ANOVA analysis was performed to evaluate the effect of the compatibilizer on the mechanical properties. Statistical differences for the measured mechanical properties were not significant.

### 2.3. SEM Assessment

**Figure 4:**
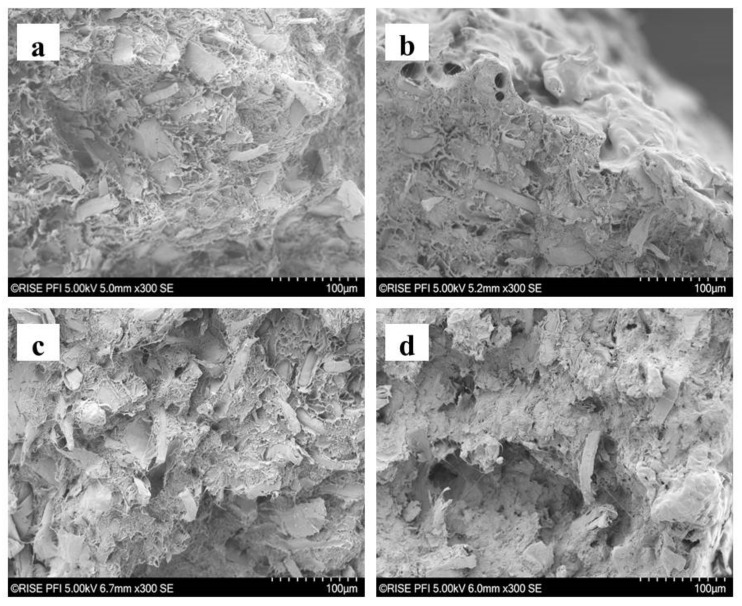
SEM microphotograph of the tensile breaking area: (**a**) 40HT-F filament, (**b**) 20HT-F filament, (**c**) 40HT-B filament, and (**d**) 20HT-B.

The SEM images of the filaments after the mechanical testing exhibit the fracture surface details. Figure 4a shows the enlarged image of the 40HT-F sample, where a large number of fibers are homogeneously distributed throughout the filament. A lower amount of fibers can be observed in the fracture area of the filament 20HT-F (Figure 4b). Besides, Figure 4b shows the high roughness of the filament surface. A similar effect with respect to fiber content was observed for the samples when a bio-based compatibilizer was used (40HT-B in Figure 4c and 20HT-B in Figure 4d). Most SEM images of the filaments showed that fibers were oriented longitudinally (0° in respect to tensile strength test direction). However, some samples presented several fibers distributed at different angles. Fiber orientation is important because it influences the final mechanical strength of filaments [32]. In addition to fiber orientation, the length of fibers is also affected by the manufacturing processes. Joffre 2014 found that fibers are drastically shortened during the manufacturing process and lose their interesting aspect ratios [33].

### 2.4. TGA and DSC

**Figure 5:**
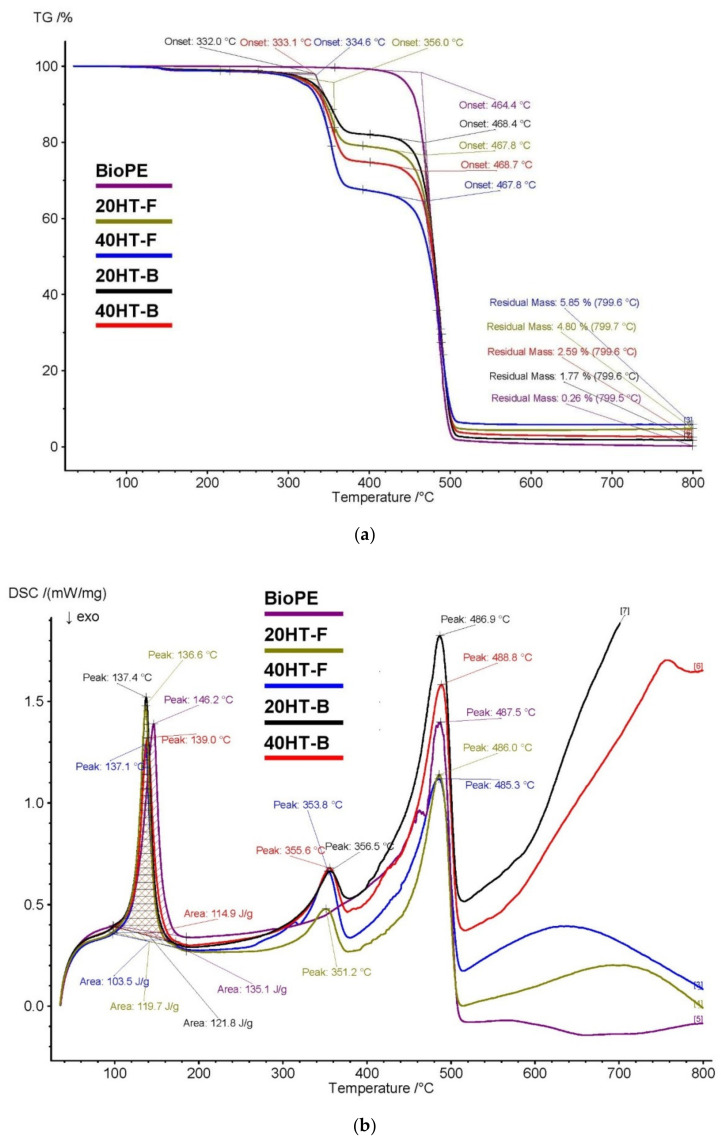
Thermo-gravimetric analysis (**a**) and differential scanning calorimetric (**b**) of the filaments.

The TGA and DSC results are shown in Table 3. The BioPE filament degraded in a single step that started at 464.4 °C as shown in Figure 5a; this process takes place rapidly and the quantity of residue is very small (0.26%). However, the BioPE composites showed two-step decomposition, where the first starts at 332–356 °C (onset temperature range), corresponding to the cellulose decomposition (around 330 °C) [34]. The second step begins at a similar onset temperature (468–469 °C), involving a fast and significant degradation attributed to the thermal cracking of the hydrocarbon chains of BioPE, which ends approximately around 510 °C [35].

The DSC thermograph is given in Figure 5b. The BioPE filament presented a first peak (146 °C) which corresponds to the melting point of the crystalline domains of the BioPE. The Tm values of all the composite samples were the same (about 130 °C) regardless of the fiber and MAPE contents. This indicated that the size of the crystalline domains, which was directly related to the Tm, was retained in the matrix. However, the melting enthalpy decreased with the increase of the fiber fraction, as expected.

The second peak can be attributed to the degradation of the hydrocarbon chains of BioPE (488 °C). The BioPE biocomposites showed three peaks. The first and third peak (137–139 °C and 485–489 °C, respectively) are associated with the melting point and degradation of BioPE [35], whilst the second peak (351−357 °C) corresponds to the thermal degradation of the fraction of bagasse fiber reinforcement, mainly due to cellulose degradation.

The addition of fibers to the matrix decreased the onset decomposition temperature of the filaments in comparison to those of pure BioPE. The weight loss at the peak around 330 °C was higher for the biocomposite filaments with high fiber contents (40%). It was lower when the bio-based compatibilizer substituted the fossil compatibilizer. This is most probably due to the different composition of the bio-based compatibilizer (97% BioPE and 2% MA), compared to the fossil-based compatibilizer (<93% fossil PE and 7% MA).

### 2.5. Printability of the Filaments

**Figure 6:**
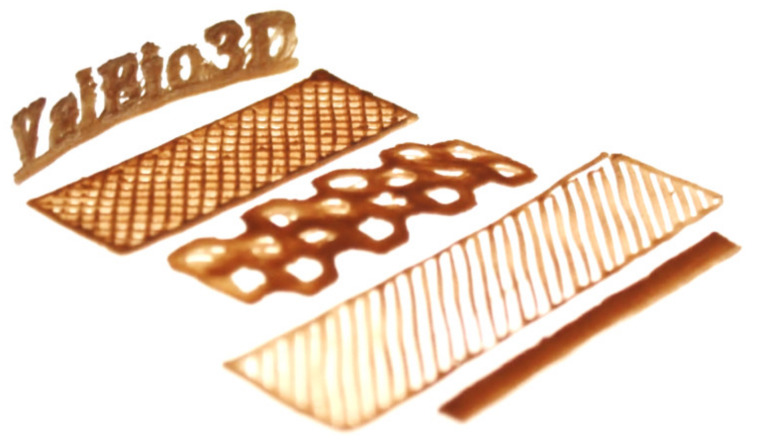
3D shapes printed with filaments containing BioPE and bagasse fibers (ValBio-3D project).

It was previously demonstrated that the addition of TMP fibers to BioPE improved printability, yielding more homogeneous structures [6]. The capability of the filaments composed of BioPE and bagasse fibers for 3D printing by FDM was demonstrated in this study (Figure 6).

Various shapes were modeled and printed, which exemplified the potential of the biocomposites for 3D printing by FDM technology. No remarkable difference was observed with respect to the printability of the different filaments, considering fiber content and type of compatibilizer (Supplementary Materials, Figure S2). It is worth emphasizing that in this study we have developed a recipe composed of 40% bagasse fibers, 54% BioPE and 6% bio-based compatibilizer. The bio-based compatibilizer was composed of 97% BioPE and only 2% MA. This implies that the biocomposite material that is printable contained less than 0.18% MA which was not bio-based. MA can also be derived from bio-based components and future initiatives could focus on developing MA from HMF, furans and furfural, that can be obtained from carbohydrates [36].

### 2.6. Environmental Aspects of Bagasse Fiber-Reinforced Biocomposites

The application of sugarcane bagasse pulp as filler in BioPE matrices for biocomposite production is mainly driven by the current need to use more environmentally friendly materials, taking into account the cost of raw materials and production processes. Thus, we performed a life cycle assessment (LCA) of bagasse fiber-reinforced BioPE pellets, comparing the environmental performance with pure sugarcane- and petroleum-based PE pellets, using as a FU 1 kg of (bio)plastic pellets. The utilization of HT bagasse fibers in a BioPE matrix decreased the effect on global warming, fossil resource scarcity, ozone formation, terrestrial acidification, and freshwater eutrophication, with respect to the use of 100% BioPE [37].

**Figure 7:**
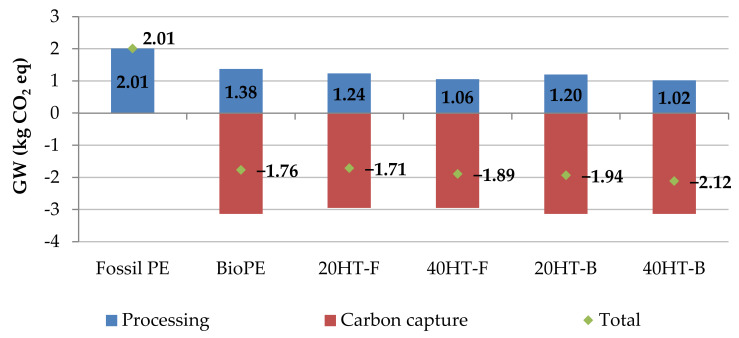
Greenhouse gas (GHG) emissions of 1 kg of fossil-based polyethylene (PE), BioPE and the corresponding biocomposites, from a cradle-to-gate perspective.

**Figure 8:**
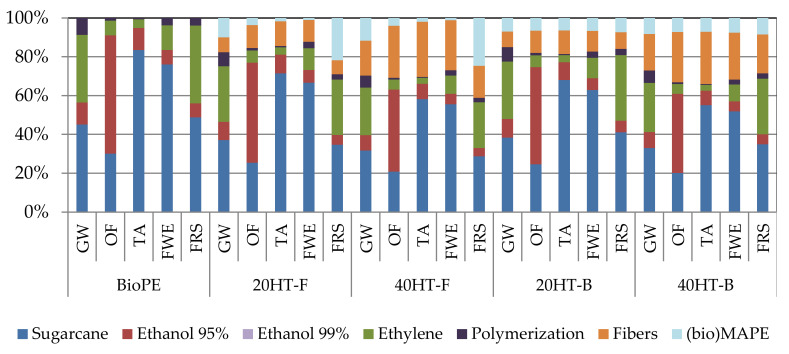
Relative contribution per life cycle stages for the production of fossil PE, BioPE and biocomposites. Results do not include carbon sequestration. Reported per functional unit: 1 kg of pellets.

In this study, we complemented the LCA considering the comparison between the bio-based and fossil compatibilizers. The results showed reductions of 3% emissions of GHG when the biocomposite uses a bio-based compatibilizer (bMAPE) rather than the fossil one (fMAPE). Additionally, when increasing the amount of fibers from 20% to 40%, reductions of GHG emissions are more notable, reaching 18%, without considering the amount of carbon storage in the final polymer. Additionally, 20HT-F, 40HT-F, 20HT-B and 40HT-B resulted in an average CO_2_-eq storage of 1.71 kg CO_2_-eq/kg, 1.89 kg CO_2_-eq/kg, 1.94 kg CO_2_-eq/kg, and 2.12 kg CO_2_-eq/kg respectively (see Table 4 and Figure 7). However, it is important to mention that this comparison is based on a replacement ratio of 1:1 between these biocomposites, which may not be the case during industrial production. Figure 8 showed that, when comparing the different stages of biocomposite production, around 40% of all impacts are related to the cultivation and harvesting stage, followed by the production of bioethylene from fermented bioethanol. Increasing the amount of fibers on the composition of the material, reductions are observed even after considering the impacts of processing raw bagasse to separate and obtain cellulose fibers. Furthermore, using a bio-based compatibilizer reduces the impacts of the production of the composite, but to a lesser extent.

When analyzing additional environmental impacts, it can be observed that, even though potential reductions of GHG emissions are obtained when utilizing bio-based MAPE instead of fossil MAPE, the environmental impact categories of ozone formation (OF), terrestrial acidification (TA) and freshwater eutrophication (FWE) show an increase of around 3% to 6%. In contrast, when comparing a biocomposite with higher fiber content, reduction on all impacts is observed.

Finally, we have demonstrated that the biocomposites developed in this study are suitable for manufacturing filaments for 3D printing. Similar recipes can be used for injection molding applications [23], which is a technology for high-volume manufacturing of e.g., automotive, furniture and packaging products.

## 3. Materials and Methods

### 3.1. Materials

Sugarcane bagasse was provided by a local mill and used in this study. A hydrothermal treatment (HT) with a liquid/bagasse ratio of 7/1, 180 °C, and 30 min at maximum temperature was performed, followed by a soda treatment using a liquid/bagasse ratio of 10/1; 170 °C, 60 min at maximum temperature, and 18% sodium hydroxide (NaOH) on oven-dry (od) bagasse (HT/Soda pulp). The determination of structural carbohydrates and lignin in biomass was carried out according to the NREL/TP–510–42618 procedure [38]. The carbohydrates were analyzed by high-performance liquid chromatography using a SHODEX SP810 (Showa Denko America, Inc., New York, NY, USA) column connected in series to a Bio-Rad (Hercules, CA, USA) deionizing pre-column. The chromatographic conditions were: water as eluent, a flow rate of 0.6 mL/min, 85 °C, and a refractive index detector. The sample was placed in a vial and frozen until the moment of analysis.

The polymer matrix was a sugarcane bio-based polyethylene (BioPE) provided by Braskem (Sao Paulo, Brazil). The BioPE had a relatively low melt flow index (MFI, 4.5 g/10 min) and a molecular weight of 92.9 g/mol.

Two compatibilizers were used to improve the compatibility between fibers and the BioPE. The fossil-based compatibilizer, maleic anhydride grafted polyethylene (denominated fMAPE), was provided in powder form by Clariant (product Licocene PE MA 4351, Clariant Plastics & Coatings (Nordic) AB, Malmö, Sweden). According to the supplier, the product is a metallocene-catalyzed PE with a grafted MA content of approx. 7 to 9 wt%. The bio-based compatibilizer was provided by YPAREX BV (Geleen, Netherlands). According to the supplier (YPAREX), the compatibilizer is derived from sugarcane BioPE with a bio-based content >97% and with a MA content <2% and was denominated bMAPE in this study.

### 3.2. Filaments Elaboration

The HT/Soda pulp was ground to a size that passed a 30 mesh sieve, and was dried for 1 h at 105 °C, whereas the BioPE and the bio-based compatibilizer were milled until passing a 10 mesh sieve.

The milled pulp fibers were assessed with a FiberTester device (L&W FiberTester Plus, Code 912. Software: Version 4.0–3.0, ABB AB/Lorentzen & Wettre, SE-164 93 Kista, Sweden). The fiber width, length and fraction of fines (objects smaller than 200 µm) were quantified. The results are based on 4037 objects that were quantified.

The pulps, BioPE, and compatibilizers were mixed as reported in Table 5. The series included two HT/Soda pulp contents (20% *w*/*w* and 40% *w*/*w*) and 6% *w*/*w* of each compatibilizer (fMAPE and bMAPE) loads. The fraction of compatibilizer (6 wt%) was selected following a previous optimization [23].

Filaments for 3D printing were manufactured as described by Filgueira et al. [18]. The blends were extruded twice in a Noztek Xcalibur filament extrusion system (Shoreham, UK). After the first extrusion the filaments were pelletized, and the pellets were used to extrude the final filament. The filament extruder had a single screw, and the filaments were extruded at a speed of 15 mm/s using three temperatures in the three sections of the extruder; 165 °C, 170 °C, and 175 °C. The speed of the extruder was determined to obtain an average filament diameter of 1.75 mm.

### 3.3. Mechanical Characterization of Filaments

Ten 60 mm length test specimens were used. The tensile mechanical properties of the filaments were measured with a Zwick/Roell (Ulm, Germany) universal tensile machine following ASTM D 5937–1996 (West Conshohocken, PE, USA). The crosshead speed was set at 100 mm/min with a 2.5 kN load cell. All tests were conducted at ambient temperature, and the means of 10 replicas were reported for each sample.

### 3.4. Water Absorption Experiments

Three filaments (60 mm) of each sample were dried for 4 h at 50 °C, and the initial weight was determined. Samples were immersed in containers with distilled water at 25 °C. After 7 days, the samples were taken out from the chambers and weighed using an analytical balance (precision of 0.1 mg). Water absorption was calculated according to the following equation:

(1) $$\mathrm{Water}\;\mathrm{absorption}\;\left(\%\right)=\frac{\left(\mathrm{Wh}-\mathrm{Wo}\right)}{\mathrm{Wo}}\times 100$$

where Wh is the weight of filaments after 7 days of immersion, and Wo is the initial weight of filaments.

### 3.5. SEM Observations

The morphology of milled fibers was assessed with scanning electron microscopy (SEM). Before SEM observation, the sample was sputter-coated with a thin layer of gold to avoid electrical charging. The applied working distance and acceleration voltage during image acquisition were 5–7 mm and 5 kV, respectively. The same settings were used to assess the fracture surfaces after tensile testing.

### 3.6. Thermo-Gravimetric Analysis (TGA) and Differential Scanning Calorimetry (DSC)

The thermal stability and decomposition temperature of the BioPE and biocomposites were determined with TGA, using Nitrogen gas, inert atmosphere, in a Netzsch Jupiter F3 equipment (Selb, Bavaria, Germany). The applied temperature range was from 30 °C to 800 °C with a heating rate of 10 °C/min. DSC experiments were performed to measure the glass transition temperature (Tg) using the same conditions and equipment.

### 3.7. 3D Printing

A REGEMAT 3D V1 printer (Granada, Spain) equipped with a 0.6 mm nozzle was used. The temperature of the nozzle was adjusted to 180 °C. The REGEMAT 3D Designer software (Version 1.0, Granada, Spain) and TinkerCad (San Francisco, CA, USA.) were used for designing the 3D models. Various shapes were printed to demonstrate the potential of the filaments in 3D printing operations.

### 3.8. Statistical Analysis

Statistical analyses were performed using the Statgraphics Centurion XV software (Statgraphics Technologies, Inc., The Plains, VA, USA). ANOVA tests were applied at a significance level *p* < 0.05.

### 3.9. Impact Assessment

An impact assessment was developed considering the production of 1 kg of biocomposite pellets as the functional unit (FU). The environmental impacts of the studied materials were analyzed using the IPCC methodology 2013 for the GHG emissions considering a 100-year period [39]. The used methodology is currently the most robust and recommended model for the estimations of global warming potential [40,41]. Additionally, considering the potential impacts related to cultivation and harvesting of biomass, impact categories related to the use of agrochemicals were also considered. The additional categories include ozone formation (OF), terrestrial acidification (TA), freshwater eutrophication (FWE) and fossil resource scarcity (FRS) at the midpoint level. These categories were evaluated using the ReCiPe methodology (Table 6). For further details on the impact assessment see Supplementary Materials and the corresponding methodology described elsewhere [42,43,44,45,46,47,48,49].

The evaluated materials in this section include a biocomposite containing 74% BioPE, 20% bagasse fibers (hydrothermal treatment) and 6% of fossil MAPE as a compatibilizer (20HT-F), a biocomposite containing 54% BioPE, 40% bagasse fibers and 6% of fossil MAPE (40HT-F), a biocomposite containing 74% BioPE, 20% bagasse fibers and 6% of bio-based MAPE (20HT-B) and a biocomposite containing 54% BioPE, 40% bagasse fibers and 6% of bio-based MAPE (40HT-B). Additionally, fossil PE and bioPE from sugarcane were also included as a baseline for comparison.

## 4. Conclusions

Filaments for 3D printing were manufactured using 100% bio-based PE, hydrothermal-soda sugarcane bagasse pulp, and bio- and fossil-based compatibilizers, demonstrating that the bagasse remaining from BioPE production can be used to obtain sugarcane bagasse pulp with adequate characteristics to reinforce BioPE, closing the loop in a biorefinery.

Increasing the fiber content caused an increase in the water absorption of the filament. The samples with 40 wt% fiber showed the highest water absorption compared to the neat BioPE filament (water absorption ~10%).

The filaments with 40 wt% fibers presented the highest tensile strengths. Increments in tensile strength of about 60% and 20% were found with fiber loads of 40 wt% and 20 wt%, respectively, using both compatibilizers. Similar behavior was found for stiffness values. The Young’s modulus reached its highest value in the filaments with 40 wt% fibers. Compared with 100% BioPE filaments, the increment in stiffness was about 75% and 25% in filaments with 40 wt% and 20 wt% fibers and both compatibilizers, respectively. Lower elongations were obtained in all cases. For all the evaluated mechanical properties, no statistically significant differences were found between filaments with fossil or bio-based compatibilizers. According to TGA analysis, the effect of compatibilizers and fiber loads on the temperature of thermal degradation is similar. TGA showed that the weight loss at the peak around 330 °C was higher for the biocomposite filaments with high fiber contents (40%).

Importantly, we demonstrated that replacing fossil compatibilizer with a bio-based compatibilizer contributes to an increase in CO_2_-capture, achieving a CO_2_-eq storage of 2.12 kg CO_2_-eq/kg for the biocomposite containing 40% bagasse fibers and 6% bio-based compatibilizer.

## Figures and Tables

**Table 1:** The chemical composition of raw material and hydrothermal treatment (HT)/Soda pulp (% oven-dried, od).

Chemical Composition (% od)	Sugarcane Bagasse	HT/Soda Pulp
Glucans	40.4 ± 0.04	90.8 ± 0.18
Hemicelluloses	35.0	5.1
Xylans	26.7 ± 0.32	5.1 ± 0.07
Arabinans	5.3 ± 0.28	-
Acetyl Groups	3.0 ± 0.02	-
Lignin	20.6 ± 0.12	2.1 ± 0.08
Extractives	3.2 ± 0.01	-

od: on oven dry base.

**Table 2:** Tensile strength (σ), Young′s modulus (E), and elongation at maximum strength (εmáx) of filaments.

Code	σ (MPa)	E (MPa)	εmáx (%)
BioPE	20.4 ± 3.1	800 ± 40	23.9 ± 2.1
20HT-F	24.8 ± 1.8	970 ± 100	18.2 ± 2.1
40HT-F	33.0 ± 2.4	1480 ± 200	21.4 ± 2.9
20HT-B	23.8 ± 3.9	1020 ± 60	19.1 ± 1.8
40HT-B	32.3 ± 2.0	1280 ± 130	19.9 ± 1.8

**Table 3:** Thermo-gravimetric analysis (TGA) and differential scanning calorimetry (DSC) analysis.

TGA Analysis				
Code	T-Onset 1 (°C)		T-Onset 2 (°C)	Residue (%)
BioPE	-		464	0.26
20HT-F	356		468	4.80
40HT-F	335		468	5.85
20HT-B	332		468	1.77
40HT-B	333		469	2.59
**DSC Analysis**				
**Code**	**Tm (°C)**	**ΔHm (J/g)**	**Tpeak 1 (°C)**	**Tpeak 2 (°C)**
BioPE	146	135	-	487
20HT-F	137	120	351	486
40HT-F	137	103	354	485
20HT-B	137	122	356	487
40HT-B	139	115	356	489

**Table 4:** Environmental burdens per impact category for evaluated materials *.

Impact Category	Unit	Fossil PE	BioPE	20HT-F	40HT-F	20HT-B	40HT-B
Global warming without CO_2_ capture	kg CO_2_ eq	2.01	1.38	1.24	1.06	1.20	1.02
Global warming with CO_2_ capture	kg CO_2_ eq	2.01	−1.76	−1.71	−1.89	−1.94	−2.12
Ozone formation, Terrestrial ecosystems	kg NO_x_ eq	4.52 × 10^−3^	8.40 × 10^−3^	7.36 × 10^−3^	6.56 × 10^−3^	7.59 × 10^−3^	6.78 × 10^−3^
Terrestrial acidification	kg SO_2_ eq	5.27 × 10^−3^	2.17 × 10^−2^	1.88 × 10^−2^	1.69 × 10^−2^	1.98 × 10^−2^	1.78 × 10^−2^
Freshwater eutrophication	kg P eq	2.79 × 10^−5^	4.80 × 10^−4^	4.05 × 10^−4^	3.55 × 10^−4^	4.30 × 10^−4^	3.80 × 10^−4^
Fossil resource scarcity	kg oil eq	1.57	0.41	0.43	0.38	0.36	0.31

* reported per functional unit: 1 kg of pellets.

**Table 5:** Biocomposites composition.

Code	BioPE (wt%)	Fibers (wt%)	Compatibilizer (wt%)	Compatibilizer Type
BioPE	100	0	0	-
20HT-F	74	20	6	fMAPE
40HT-F	54	40	6	fMAPE
20HT-B	74	20	6	bMAPE
40HT-B	54	40	6	bMAPE

**Table 6:** Environmental impact categories considered for the environmental assessment.

Methodology	Impact Category	Unit
IPCC 2013	Global warming 100 year (GW)	kg CO2 eq
ReCiPe 2016	Ozone formation, terrestrial ecosystems (OF)	kg NOx eq
	Terrestrial acidification (TA)	kg SO2 eq
	Freshwater eutrophication (FWE)	kg P eq
	Fossil resource scarcity (FRS)	kg oil eq

## Supplementary Materials

The following are available online. Figure S1: Bagasse fibres, fracture areas of filaments (bagasse fibres and BioPE) and 3D printed tracks; Figure S2: Optical images of part of 3D printed layers. The images were acquired with an Epson Perfection scanner in transmission mode, 4800 dots per inch. From left to right) 20HT-F, 40HT-F, 20HT-B and 40HT-B; Figure S3: Graphical representation of the system boundaries for the production of sugarcane bioPE and fiber reinforced biocomposites. * Hemicelluloses are a by-product of the bagasse hydrothermal treatment.
