# Supplementary material for: Biocomposites of Bio-Polyethylene Reinforced with a Hydrothermal-Alkaline Sugarcane Bagasse Pulp and Coupled with a Bio-Based Compatibilizer
Source: Molecules. 2020 May 5;25(9):2158. doi: 10.3390/molecules25092158 (PMC7249044; doi:10.3390/molecules25092158)
Supplement: Supplementary file 1 [file molecules-25-02158-s001.pdf]

Supplementary Materials

Fibers, Filaments and 3D Printed Structures

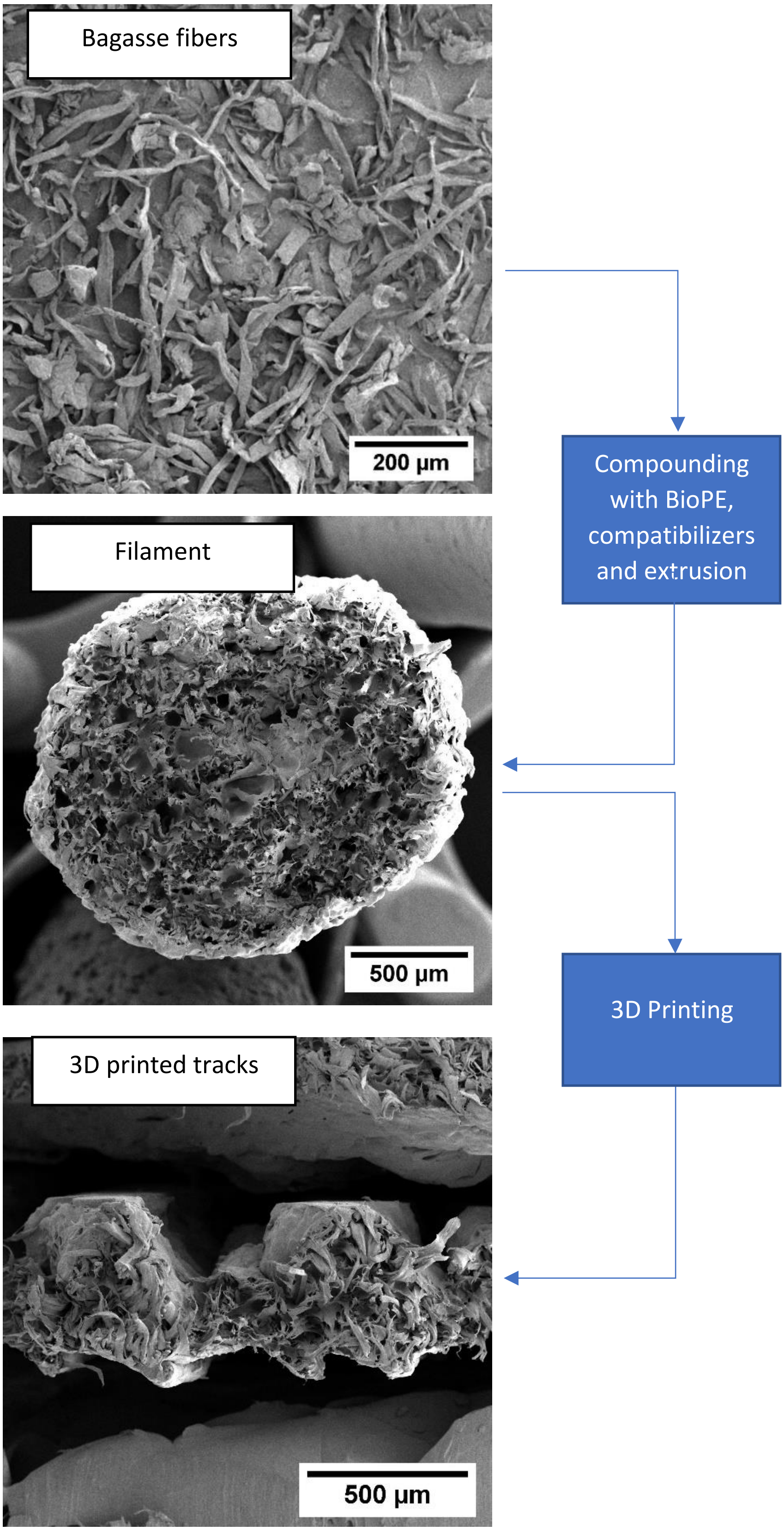

Figure S1. Bagasse fibers, fracture areas of filaments (bagasse fibers and BioPE) and 3D printed tracks.

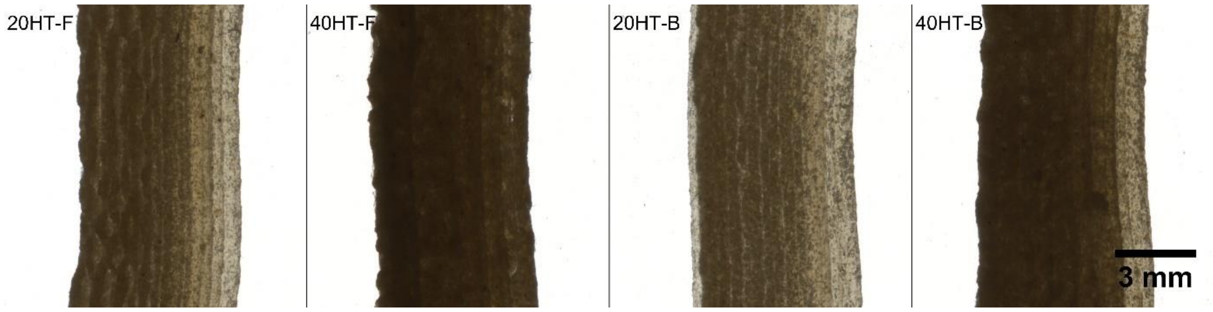

Figure S2. Optical images of part of 3D printed layers. The images were acquired with an Epson Perfection scanner in transmission mode, 4800 dots per inch. From left to right) 20HT-F, 40HT-F, 20HT-B and 40HT-B. Note the darker color of samples 40HT-F and 40HT-B, which is due to the larger fraction of fibers (40%).

Analytical Framework of the Life Cycle Assessment

Life Cycle Assessment (LCA) is a commonly used methodology to evaluate the potential environmental implications of a product or service throughout its life cycle. The main benefit of this method is obtaining a full picture of all the processes, inputs and outputs (i.e. materials, energy and emissions) while mapping environmental impacts and identifying possible problem shifting to upstream or downstream phases [42,43]. The LCA methodology is broadly described in a set of standards specified in the ISO 14040 and 14044 guidelines [44,45]. These ISO standards describe the main principles and framework of an LCA, which include four phases: the definition the goal and scope, the life cycle inventory analysis (LCI), the life cycle impact assessment (LCIA) and the interpretation of the results obtained [46].

These studies can be performed considering different scopes, which determine the processes included during the assessment. During the present evaluation, a cradle-to-gate scope was considered. A cradle-to-gate study starts with the processes of extraction of raw material and ends when the evaluated product leaves the factory gate [47]. Thus, final disposal and processes after the disposal (landfilling, recycling, incineration) were excluded from the evaluation.

The system boundaries for the study, included the extraction of (biobased) raw materials, including processes of harvesting and cultivation, processing of materials and the obtaintion of bagasse-based fibers to be used as fillers during the production of the biocomposites. The last step was the pellet production through polimerization. Figure 3 shows the system boundaries of the study.

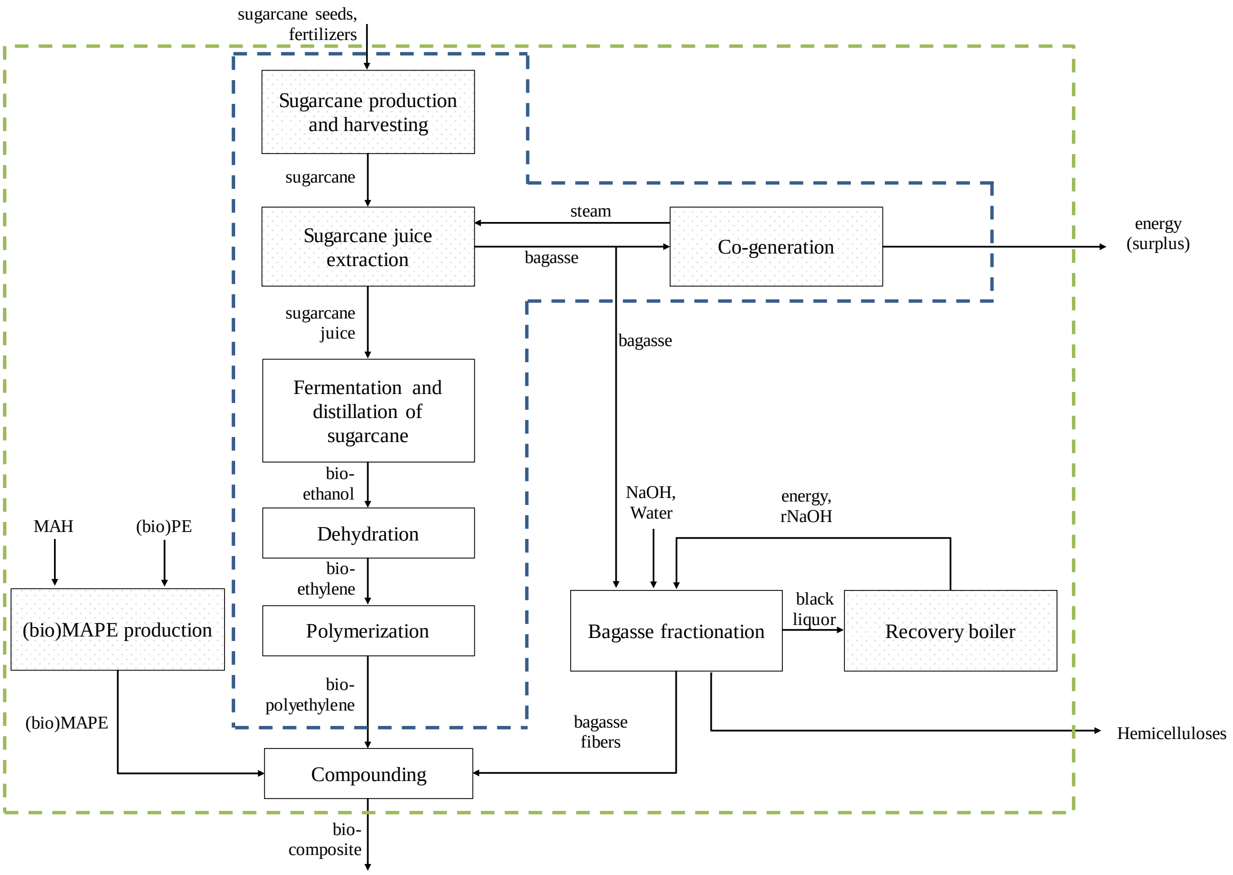

Figure S3. (Adapted from [37]). Graphical representation of the system boundaries for the production of sugarcane bioPE and fiber reinforced biocomposites. * Hemicelluloses are a by-product of the bagasse hydrothermal treatment.

The functional unit (FU) describes the main function of the system for example, one cultivated hectare, one kilometer, one produced tonne and the environmental impacts are expressed by functional unit. During this assessment, the FU was defined as 1 kg of plastic or composite pellets ready to be used.

For the modelling of the system, data available in Ecoinvent v3.4 database was used as background data and for the processes of sugarcane production and harvesting, and energy generation (assumed to be provided by Brazilian national grid) [48]. The information for the elaboration of bioethanol, bioethylene, MAPE and bioMAPE was retrieved from producers through direct communication. Finally, data for the fractionation of bagasse was obtained from laboratory experiments performed at the Institute of Materials at Misiones, Argentina. The software SimaPro 8.5.2.0 [49], developed by PRé Sustainability, was used to perform the LCA calculations.
